# Potential underutilisation of weight loss medications in obese patients with knee osteoarthritis

**DOI:** 10.1308/rcsann.2026.0063

**Published:** 2026-08-06

**Authors:** N Badhe, C Busby, T Kurien, BA Marson

**Affiliations:** ^1^ University of Nottingham, UK; ^2^ University of Cambridge, UK

**Keywords:** Obesity, Knee osteoarthritis, GLP-1 receptor agonists, BMI thresholds, Barriers to surgery

## Abstract

**Introduction:**

Obesity is a major risk factor for knee osteoarthritis (OA), contributing to earlier symptomatic onset, increased pain severity and greater lifetime disability. Despite guidance that weight alone should not limit access to total knee arthroplasty (TKA), body mass index (BMI) thresholds are applied widely in clinical practice. This study investigated the glucagon-like peptide-1 (GLP-1) receptor agonist (RA) use among obese OA patients awaiting TKA and evaluated the influence of BMI on access to TKA.

**Methods:**

A 30% random sample of a single-centre TKA waiting list (*n* = 300) in August 2025 was analysed. Data extracted included age, sex, BMI, time on waiting list, surgical listing status, comorbidities and use of weight loss interventions. Patients were stratified by BMI category (<35, 35–40, >40) and sex.

**Results:**

Median BMI was 33.0 [27.9–38.0], with 120 patients (40%) classified as obese (BMI ≥35). Among obese patients, 45% were initially denied or conditionally listed for surgery, rising to 74.1% for BMI >40. Only 13 obese patients (10.8%) were taking GLP-1 RAs, despite extensive eligibility. Female patients had a higher average BMI (*p* = 0.049) and faced higher denial rates than male patients (52% vs 28%, *p* = 0.013). Most obese patients (*n* = 102) had ≥3 comorbidities, often including diabetes (*n* = 22) and obstructive sleep apnoea (*n* = 26).

**Conclusion:**

Current pathways for managing obese patients with knee OA are inadequate. GLP-1 RAs may offer a promising solution to help reduce barriers to surgery. Further research is needed to determine whether GLP-1 RAs could help reduce the progression of OA and need for TKA.

## Introduction

Obesity is the most important modifiable risk factor for knee osteoarthritis (OA), driving both disease onset and symptom progression.^1^ Obese patients therefore present with symptomatic OA at a younger age, report more severe pain, and experience greater lifetime disability.^2,3^ However, obese patients face systemic barriers to total knee arthroplasty (TKA) surgery, with body mass index (BMI) eligibility thresholds utilised widely in clinical practice. This is despite national guidance that weight alone should not restrict access to knee arthroplasty.^4^

Glucagon-like peptide-1 (GLP-1) receptor agonists (RAs) have emerged as an effective pharmacological intervention for weight management, demonstrating significant and reliable weight loss in randomised controlled trials (RCTs).^5,6^ Since gaining NHS approval for the management of type 2 diabetes mellitus (T2DM) and for patients with a BMI >35 who are significantly affected by obesity-related conditions, GLP-1 RAs have stimulated interest among arthroplasty surgeons for the management of obese patients with lower limb OA.^7–9^

In addition to promoting weight loss, recent evidence indicates GLP-1 RAs improve glycaemic control,^10^ decrease cardiovascular risk and may provide a direct chondroprotective effect.^11–13^ For obese, often comorbid, OA patients, GLP-1 RAs may help to reduce BMI to below surgical eligibility thresholds, while simultaneously reducing cartilage degeneration and improving comorbidity burden. In our recent systemic review, we found that GLP-1 RAs may reduce pain and improve physical function in obese patients with lower limb OA (Badhe *et al*, unpublished). However, these findings were uncertain due to limited RCT evidence. GLP-1 RAs may therefore present a promising therapeutic opportunity; however, the current uptake of GLP-1 RAs among obese OA patients remains unknown.

Despite the growing clinical potential of GLP-1 RAs, their inclusion in current pathways remains limited. This study aimed to determine the current utilisation of GLP-1 RAs among obese knee OA patients, evaluate how BMI influences TKA listing decisions, and identify deficiencies in existing management pathways.

## Methods

A single-centre, cross-sectional analysis of knee OA patients listed for primary TKA at a large NHS teaching hospital was conducted. A randomised sample of 30% of the active TKA waiting list was selected to ensure it was representative of the overall waiting list. All patients with a diagnosis of knee OA listed for primary TKA as of August 2025 were eligible for inclusion. Patients undergoing revision surgery or knee arthroplasty for post-traumatic or inflammatory arthritis were excluded. For each included patient, demographic information, clinical characteristics and surgical listing status were extracted from electronic health records and preoperative assessment documentation.

The data collected for each included patient included:

• Demographics – age and sex
• BMI – as recorded at the preoperative assessment
• Surgical listing status – measured using NHS England surgical prioritisation categories 1–4. Priority 1 – procedures to be performed in <72 hours; Priority 2 – procedures to be performed in less than one month; Priority 3 – procedures to be performed in less than three months; Priority 4 – procedures to be performed after more than three months.
• Total time on the waiting list

Additionally, the following information was collected for all included patients with BMI >35kg/m^2^:

• The proportion of patients that were initially denied or conditionally listed for surgery due to high BMI. Patients were ‘denied’ if they were not listed at the time of assessment due to BMI, and ‘conditionally listed’ if they were listed contingent on achieving weight loss preoperatively.
• Eligibility for GLP-1 RA, as per NHS and National Institute for Health and Care Excellence (NICE) guidance.^14,15^
• Current GLP-1 RAs use.
• Use of alternative pharmacological weight loss therapy, participation in commercial weight loss clubs, or history of bariatric surgery.
• The proportion of patients with three or more comorbidities, and specifically the proportion of patients affected by significant cardiovascular or respiratory disease, T2DM, and obstructive sleep apnoea (OSA).
• The proportion of patients who had been transferred from non-NHS hospitals due to high BMI

Patients were stratified by BMI (<35, 35–40 and >40kg/m^2^) for the primary analysis. An exploratory subgroup analysis stratifying by sex was conducted for patients with BMI ≥35kg/m^2^ to explore gender differences in access to surgery.

Data were analysed using descriptive statistics. Continuous variables were nonparametric and therefore presented as medians with interquartile range, whereas categorical variables were presented as frequencies and proportions. Differences between BMI groups were assessed using Mann–Whitney *U* tests for continuous variables and chi-square tests for categorical variables. A significance threshold of *p*<0.05 was used throughout the analysis. Statistical analyses were performed using GraphPad Prism version 10.5.1.

## Results

A total of 300 patients were included in the final analysis. The median age [range] of included patients was 68.0 years [61.8–78.0] and 166 (55.3%) were female. Median BMI was 33.0 [27.9–38.0], with 120 (40%) patients classified as obese (BMI ≥35kg/m^2^). Patients with BMI ≥35 were younger (62.5 vs 73.0 years, *p* < 0.001) and more likely to be female (70% vs 46%, *p* < 0.001).

### Surgical listing status

When stratified by BMI, 180 patients (60%) had BMI <35, 66 (22%) had BMI 35–40 and 54 (18%) had BMI >40. There were no significant differences in surgical listing status across BMI groups, with 135 (75.0%), 53 (80.3%) and 38 (70.4%) patients listed as Priority 4, respectively (*p* = 0.448). Similarly, time on the waiting list did not differ significantly between BMI groups: 256 days [254–386] for BMI <35 vs 223 days [168–350] for BMI 35–40 vs 229 days [170–433] for BMI >40 (Table 1).

**Table 1:** Demographics, surgical listing status and weight loss interventions in TKA waiting list patients stratified by BMI.

Variable	BMI <35	BMI 35-40	BMI >40
Total	180	66	54
Median age, years [range]	73 [64–79]	63.5 [59–75]	62 [59–68.75]
Sex (M:F)	98M: 82F	26M:40F	10M:44F
Median BMI, kg/m^2^ [range]	29.05 [26.28–31.55]	37.1 [36.1–38.18]	43.5 [41.35–46.23]
Priority 1: procedures to be performed within less than 72 hours, *n* (%)	0 (0)	0 (0)	0 (0)
Priority 2: procedures to be performed within less than one month, *n* (%)	12 (6.67)	1 (1.51)	5 (9.26)
Priority 3: procedures to be performed within less than three months, *n* (%)	33 (18.33)	12 (18.18)	11 (20.37)
Priority 4: procedures to be performed after three months, *n* (%)	135 (75)	53 (80.30)	38 (70.37)
Median time on waiting list, days [range]	256 [153.75–385.50]	223 [168–350.25]	229 [170–432.75]
Currently taking GLP-1 RA, *n* (%)	n/a	7 (10.61)	6 (11.11)
Initially denied/conditional listing, *n* (%)	n/a	14 (21.21)	40 (74.07)
Weight loss target n/s	n/a	11 (78.57)	20 (50)
Weight loss target BMI <40, *n* (%)	n/a	3 (21.43)	16 (40)
Weight loss target BMI<35, *n* (%)	n/a	0 (0)	4 (10)
Transferred from non-NHS hospitals	n/a	3 (4.55)	7 (12.96)
Active weight loss club, *n* (%)	n/a	1 (1.51)	5 (9.26)
Currently taking other weight loss drug, *n* (%)	n/a	1 (1.51)	0 (0)
Previous bariatric surgery, *n* (%)	n/a	1 (1.51)	1 (1.85)

BMI = body mass index; F = female; GLP-1 = glucagon-like peptide-1; M = male; n/a = not applicable; n/s = not specified; RA = receptor agonist; TKA = total knee arthroplasty

### Access to TKA

Among the 66 patients with BMI >35, 54 (45%) were initially denied or conditionally listed for surgery. This figure rises to 74.1% for patients with BMI >40 (*p* < 0.001). Weight loss thresholds for these patients were applied inconsistently, with only three (21.4%) patients with BMI 35–40 and 20 (50.0%) patients with BMI >40 provided specific BMI targets when initially denied or conditionally listed. An exploratory subgroup analysis stratifying the 120 knee OA patients with BMI >35 by sex demonstrated that 44 (52.4%) obese female patients were initially denied or conditionally listed compared with 10 (27.8%) obese male patients (*p* = 0.013). However, obese female patients included in this analysis also presented with a higher median BMI (40.3 vs 38.1; *p* = 0.049).

### Utilisation of weight loss interventions

GLP-1 RA use was recorded in thirteen obese patients (10.8%), with only two patients (1.7%) were contraindicated to the therapy. Uptake of other weight loss interventions was also limited, with just six patients (5.0%) attending a commercial weight loss club such as Weight Watchers or Slimming World, one patient (0.8%) receiving an alternative pharmacological therapy for weight loss and two patients (1.7%) undergoing previous bariatric surgery.

### Comorbidity burden and transfer from non-NHS hospitals

Nearly all obese patients had comorbid disease, with 102 (85%) patients having three or more comorbidities and significant cardiovascular or respiratory disease present in 40 (33.3%) patients. Notably, T2DM and OSA affected 22 (18%) and 26 (22%) of obese knee OA patients, respectively. Further analysis demonstrated that ten patients (8.3% of all obese patients) were denied surgery in non-NHS hospitals due to high BMI.

## Discussion

These findings highlight the inadequacies of current pathways for the management of obese patients with knee OA and demonstrate that clinically implemented BMI-related barriers often lead to inequity in access to surgery. Almost 50% of all patients with BMI ≥35kg/m^2^ were initially denied or conditionally listed for TKA, despite national guidance advising that weight alone should not limit access to surgery. Currently, only 10% of obese OA patients are taking GLP-1 RAs. GLP-1 RAs have been shown to provide rapid and reliable weight loss.^5,6^ GLP-1 RAs therefore offer a promising solution to enable obese patients to meet clinically implemented BMI thresholds, reduce barriers to TKA and minimise inequity in surgical pathways.

The analysis identified no significant difference in surgical listing priority or time on the waiting list. However, these analyses did not account for initial delays that obese knee OA patients face before arthroplasty listing. It is also important to note that even when patients were listed for TKA subject to weight loss, active weight loss programs with specified BMI targets were applied infrequently and inconsistently, varying between the arbitrary thresholds of <40 or <35kg/m^2^. Such heterogeneity in clinical practice risks leaving patients unsupported, with no reliable pathway to TKA eligibility. Targeted administration of GLP-1 RAs could provide an effective and scalable strategy that enables patients to meet agreed clinical thresholds rapidly and reliably.

A disproportional impact of obesity on surgical thresholds for female patients was identified.^16^ Obese women were initially denied or conditionally listed more frequently compared with obese men. However, this analysis was confounded by women presenting with higher BMI and also does not take into account variation in the distribution of adipose tissue, which may affect surgical complexity in TKA.^17^ These findings indicate that clinically utilised BMI thresholds may also contribute to gender-based inequities regarding access to TKA.^18^

Weight loss is demanded frequently for TKA eligibility, but patients are rarely provided with an effective means to achieve these, often unspecified, targets. Additionally, obese patients with knee OA were often comorbid, with high rates of T2DM and OSA. Both conditions not only increase surgical risk but also demonstrate improvement with GLP-1 RA administration.^10,19^ Consequently, the observed discrepancy between minimal GLP-1 RA uptake despite extensive eligibility may represent a missed opportunity. However, this study could not investigate the reasons underlying the relatively low uptake of GLP-1 RAs. Consequently, factors that may influence uptake including access to GLP-1 RA, clinician familiarity and patient preference should be investigated in future studies.

Transfer of a patient’s planned surgical care from non-NHS hospitals affected almost 10% of all obese patients. Targeted use of GLP-1 RAs could therefore enhance access to surgery across healthcare settings and potentially reduce the need for such transfers, which often result in significant delays in time to surgery for these patients.

## Proposed mechanisms

The benefit of GLP-1 RA-mediated weight loss on reducing knee OA pain through reducing mechanical knee joint load is clear.^20^ In addition, the decrease in adipocytokines and other inflammatory metabolites and direct chondroprotective effects may reduce knee OA.^21–27^

GLP-1 RAs may therefore offer significant benefits for obese patients with knee OA, reducing the progression of joint degeneration and, in some patients, even eliminate the need for TKA. Analysis of registry data by Yang *et al* and Porto *et al* found that GLP-1 RA use was associated with a reduced risk of progression to TKA.^28,29^ Similarly, a prospective cohort study by Zhu *et al* reported that obese knee OA patients taking GLP-1 RA were less likely to require future knee surgery compared with controls.^13^ Conversely, registry data studies by Lavu *et al* and Porto *et al* found that patients taking GLP-1 RAs had a significantly higher risk of developing knee OA^29,30^ and Bliddal *et al*, the only RCT to investigate the effect of GLP-1 RAs on the progression of OA to needing TKA, reported a slightly higher, but not statistically significant, rate of TKA in patients taking GLP-1 RA compared with placebo.^9^ However, these findings are likely influenced by the fact that GLP-1 RA-mediated weight loss makes patients more appealing candidates for TKA.

To date only two randomised trials have investigated role of GLP-1 RAs for knee OA.^9,31^ The recent Bliddal *et al* trial reported significant improvements in both WOMAC pain (−41.7 vs −27.5, *p* < 0.001) and SF-36 physical function change scores (12 vs 6.5, *p* < 0.001) with patients treated with GLP-1 RA compared with placebo. However, these findings were not observed in the earlier Gudbergsen *et al* trial. This may be due to a critical difference in the study design between these two RCTs. Participants were only recruited to the Gudbergsen *et al* trial if they achieved >5% weight loss in an 8-week dietary intervention, reducing the generalisability of the findings of this trial to patients capable of achieving weight loss through dietary intervention. Despite the limited evidence, the Bliddal *et al* trial demonstrates promising outcomes; however, it is critical to recognise that both trials were funded by Novo Nordisk—a manufacturer of multiple brands of GLP-1 RAs.

We therefore suggest an independent, large, multicentre RCT investigating the role of GLP-1 RAs compared with standard rehabilitation pathways for the management of obese knee OA patients, with early intervention at the point of referral to an arthroplasty surgeon and extended follow-up. Such a trial could provide the necessary evidence on its efficacy, safety and impact on progression of OA and need for future TKA to transform current practice. Until then, thousands of patients will continue to experience prolonged pain and disability while a potentially effective and available therapy remains underutilised.

## Conclusion

This study demonstrates the inadequacy of current pathways for the management of obese knee OA patients. Knee OA patients with obesity are systematically disadvantaged in access to TKA, with higher denial rates, conditional listing and inconsistent application of weight loss targets. Currently only 10% obese OA patients are taking GLP-1 RAs. This represents a missed opportunity. GLP-1 RAs could help obese OA patients meet the BMI thresholds commonly implemented in clinical practice for TKA eligibility. However, an independent, large, multicentre RCT evaluating the use of GLP-1 RAs at the point of referral to an arthroplasty surgeon is required to provide the robust evidence required to overcome barriers to surgery and revolutionise current practice.

## Competing interests

The author/s declare no competing interests.

## Funding

The author/s received no financial support for the research, authorship and/or publication of this article.

## Ethics approval and consent to participate

This study was registered as a service evaluation with Nottingham University Hospitals NHS Trust (audit registration number 25-301C).

## Author contributions

**N Badhe:** Data curation, Formal analysis, Investigation, Methodology, Writing – original draft, Writing – review & editing. **C Busby:** Data curation, Formal analysis, Investigation, Methodology, Supervision, Validation, Visualization, Writing – original draft, Writing – review & editing. **T Kurien:** Investigation, Methodology, Project administration, Supervision, Validation, Visualization, Writing – original draft, Writing – review & editing. **B Marson:** Conceptualization, Data curation, Formal analysis, Investigation, Methodology, Project administration, Supervision, Validation, Visualization, Writing – review & editing.

## Artificial Intelligence

The author/s declare that no AI was used to conduct the study or prepare the manuscript.
